# The Effect of Keppra Prophylaxis on the Incidence of Early Onset, Post-traumatic Brain Injury Seizures

**DOI:** 10.7759/cureus.2674

**Published:** 2018-05-23

**Authors:** Ali Hazama, Robert Ziechmann, Manu Arul, Satish Krishnamurthy, Michael Galgano, Lawrence S. Chin

**Affiliations:** 1 Neurosurgery, SUNY Upstate Medical University, Syracuse, USA; 2 School of Medicine, SUNY Upstate Medical University, Syracuse, USA

**Keywords:** traumatic brain injury (tbi), seizure prophylaxis, keppra, post-traumatic epilepsy

## Abstract

Objective: Traumatic brain injury (TBI) is a leading cause of long-term disability. Early onset post-traumatic seizures (PTS) after traumatic injury to the brain is a strong predictor of adverse outcomes in these patients. Our study investigates the role of Keppra in early PTS prophylaxis compared to no treatment, taking into account risk factors including injury severity, seizure history, and anti-epileptic drug (AED) use.

Methods: This was a retrospective cohort study based on patient chart data from January 2013 to January 2017 at a level one trauma center in the United States. A t-test was performed with P<0.05 as significant; we utilized a 95% confidence interval (CI) for our findings. Subgroup analysis was performed, with respect to the Glasgow Coma Scale (GCS) score (Group A: Mild GCS=13-15, Keppra N=135, Non-Keppra N=122; Group B: Moderate GCS=9-12, Keppra N=23, Non-Keppra N=19; Group C: Severe GCS= <8, Keppra N=69, Non-Keppra=35).

Results: Of 403 patients included in the study, 227 were given Keppra. Demographics between treatment groups were similar. Whole cohort analysis confirmed six patients with PTS, and no significant difference between groups (Keppra N=3, Non-Keppra N=3, OR=0.77, P=0.75, 95% CI=(0.154-3.87)). Subgroup analysis revealed reduction in seizure incidence in Keppra groups A (OR=0.18, P=0.27, 95% CI=(0.008-3.80)) and B (OR=0.82, P=0.92, 95% CI=(0.015-43.7)), but this reduction was not statistically significant. Those with the severe TBI in group C accounted for the majority of seizures (n=4, OR=1.52, P=0.71, 95% CI=(0.15-15.4)).

Conclusion: Patients with more severe TBI suffered a higher incidence of early-onset post-traumatic seizures. Data of the cohort as a whole revealed a trend towards a lower seizure incidence in patients who were treated with Keppra prophylaxis. Despite this trend, the decrease in seizure incidence did not reach statistical significance.

## Introduction

Traumatic brain injury (TBI) is a leading cause of long-term disability, and an estimated 3.3 to 5.3 million people live with some degree of impairment from TBI in the United States [1]. Seizures are one of the major sources of impairment after patient endures a TBI. Early post-traumatic seizures (PTS), defined as seizures within one week of admission, are considered to be a strong predictor of late PTS and epilepsy [2]. Early PTS is also associated with higher rates of pneumonia, acute respiratory distress syndrome (ARDS), acute renal failure, pulmonary embolism, and increased intracranial pressure (ICP). However, there is significant overlap in the risk factors for early seizure and many of these complications, therefore, the relationship between early PTS and patient outcomes is controversial. Risk of early post-traumatic seizure increases with a Glasgow Coma Scale (GCS) score of ≤ 10; subdural, epidural, or intracranial hematoma; linear or depressed skull fracture; cortical contusion; and age ≤ 65 years [3].

The current guidelines from the Brain Trauma Foundation recommend phenytoin (Dilantin) prophylaxis to reduce the rate of early PTS [4]. At the time of publication, the authors of these guidelines noted that there is insufficient evidence to recommend levetiracetam (Keppra) over Dilantin prophylaxis. According to a recent systematic review and meta-analysis of 11 randomized controlled trials and controlled observational cohort studies, Keppra was not superior in efficacy compared to phenytoin in terms of early or late PTS incidence [5]. There is less data comparing Keppra to no treatment or placebo. To date, only one retrospective study has directly compared the rate of early PTS in those receiving Keppra prophylaxis versus no treatment, finding a non-significant decrease from 3.4% to 1.9% in the prophylaxis group [6].

The purpose of our study was to compare the rate of early PTS with Keppra prophylaxis versus no treatment, taking into account the risk factors for early PTS as mentioned above, as well as individual seizure history, anti-epileptic drug (AED) use, and severity of the injury.

## Materials and methods

This was a retrospective cohort study based on patient data from January 2013 to January 2017 at a level one trauma center in the United States. After obtaining approval from the SUNY Upstate Medical University Institutional Review Board, patients who were admitted to the hospital with TBI were identified by searching the billing record via Current Procedural Terminology (CPT) codes. There is no specific protocol for seizure prophylaxis after TBI at our institution, and the heterogeneity in physician preference in terms of using seizure prophylaxis or not provided us with two groups of patients: those who received seizure prophylaxis and those who did not. The physicians in our group who prescribe seizure prophylaxis uniformly utilized Keppra. Chart review was done to collect data on patient demographics, neurologic status at the time of admission, mechanism of injury, initial imaging findings, anti-epileptic therapy, seizure history, and adverse drug reactions. The primary endpoint of our study was early post-traumatic seizure (seizure within one week of admission).

Patients were divided into three groups based on the severity of the TBI sustained. Groups A, B, and C included patients with GCS score of less than or equal to eight, between 9-12, and 13-15 respectively (Table 1). A T-test was used to calculate a t-value from which a p-value was derived. A p-value of <0.05 was used to establish statistical significance and a 95% confidence interval (CI) was calculated for the primary outcome of the study for the entire cohort as well as subgroup analysis for each injury severity group.

**Table 1:** Patients were divided into three groups A, B, and C based on the severity of their injury as calculated by the GCS score at presentation GCS: Glasgow Coma Scale.

Severity of Injury			
Group	GCS	Keppra group	Non-Keppra Group
A	Mild (GCS =13-15)	135	122
B	Moderate (GCS = 9-12)	23	19
C	Severe (GCS ≤ 8)	69	35

## Results

A total of 471 TBI patients were initially identified in our database between January 2013 and January 2017. Of these, 68 were excluded due to age < 18, history of epilepsy, and mortality. Of the 403 patients included in our study, 227 had been given Keppra prophylaxis and 176 did not receive seizure prophylaxis. Patient demographics were similar between the two groups with a mean age of 32 in the Keppra group and 33 in the non-prophylaxis group. Both groups were predominantly male (76% versus 67%). Mechanisms of injury were similar in the two groups, with automobile accidents being the most common cause of TBI (Table 2). The most common initial finding on computed tomography (CT) of the head was traumatic subarachnoid hemorrhage (tSAH), followed by subdural hematoma (SDH) and skull fracture.

**Table 2:** Demographics, mechanism of injury, and initial head computed tomography (CT) findings on presentation in the seizure prophylaxis and non-prophylaxis groups

Demographic, mechanism of injury, and initial head CT findings		
	Keppra group	Non-Keppra Group
Age	32 ± 16	33 ± 15
Sex (% male)	76	67
Automobile Accident	125	103
Assault	47	33
Bicycle Accident	17	15
All-Terrain Vehicle (ATV)	17	11
Fall	13	9
Motorcycle Accident	8	4
Other	0	4
tSAH	100	48
SDH	84	53
Skull Fracture	64	50
Contusion	40	23
IPH	29	13
EDH	16	11
IVH	15	13

The initial analysis was performed on the cohort as a whole, and revealed a total of six patients who had developed seizures within the first seven days of admission. Interestingly, three seizures occurred in the Keppra group and thee in the control group. A trend towards decreased seizure incidence was observed in the Keppra group of 1.36% vs. 1.70% in the control group. Statistical analysis revealed no significant difference in the observed seizures in the treated group versus the group without Keppra prophylaxis (p-value 0.75).

Further subgroup analysis revealed similar results. Group C which included patients with severe TBI accounted for the majority of seizures (n=4). Less seizures were observed in both groups A and B (Table 3). Patients with moderate and severe injuries were pooled together and further analysis was performed. In this subgroup, a total of three seizures occurred in the Keppra group, while only one was reported in the control group. The Keppra group was not found to be significantly superior to the group without prophylaxis.

**Table 3:** Seizure incidence in the Keppra and non-Keppra groups Odds ratios, confidence intervals, and P-values are also reported here.

Seizure Incidence and statistical analysis					
	Keppra Group	Non-Keppra Group	Odds ratio	Confidence Interval	P-Value
All groups	3 (1.36%)	3 (1.70%)	0.77	0.154 to 3.87	0.75
Group A	0 (0.00%)	2 (1.63%)	0.18	0.008 to 3.80	0.27
Group B	0 (0.00%)	0 (0.00%)	0.82	0.015 to 43.7	0.92
Group C	3 (4.48%)	1 (2.86%)	1.52	0.15 to 15.4	0.71
Groups B and C	3 (3.26%)	1 (1.85%)	2.41	0.24 to 23.8	0.45

## Discussion

The use of Keppra for early seizure prophylaxis in traumatic brain injury has been debated in the literature for over a decade. In 2008, Jones et al. suggested Keppra as an alternative to phenytoin, citing the potential advantages of fewer drug-drug interactions and no need for serum monitoring [5]. This initial prospective cohort study of 73 patients found an equivalent seizure rate for those with severe TBI (GCS ≤ 8) on Keppra to patients receiving phenytoin prophylaxis. The authors did, however, note a trend toward epileptiform activity on electroencephalogram (EEG) for those receiving Keppra. Subsequent studies have had mixed results in terms of both efficacy of seizure prophylaxis and overall clinical outcomes.

In terms of efficacy, only one retrospective observational study of 109 patients with severe TBI showed a lower seizure rate with Keppra versus phenytoin prophylaxis [7]. The trend in our sub-group analysis for severe TBI shows instead a higher seizure rate with Keppra prophylaxis, although our results did not approach statistical significance. A larger series of patients (n = 813) with severe TBI or mild to moderate TBI with an abnormal head CT found no difference in seizure rate [3]. Similarly, there was no difference in seizure rate in a randomized controlled trial comparing Keppra and phenytoin for patients with severe TBI [8]. No difference was seen in several other prospective and retrospective cohort studies that also included patients with mild to moderate TBI [3-4,9-10]. As previously mentioned, Keppra has only been compared to no treatment in one study previous to our cohort. This retrospective study showed a slightly lower rate of seizures in the severe TBI group (1.9% versus 3.4%), but no statistically significant difference with levetiracetam [11].

The overall clinical outcome of patients on Keppra versus phenytoin prophylaxis is unclear [8,12-15]. A prospective randomized study of 52 patients found a lower Disability Ratings Scale at three months and a higher Glasgow Outcome Score at six months for those on Keppra prophylaxis compared to phenytoin [12]. Another study (n = 19) found no difference in the Glasgow Outcome Scale-Extended at six months [2]. Inaba et al. (n = 813) also found no difference in terms of adverse drug reactions or mortality between those on Keppra and phenytoin prophylaxis [3]. A systematic review of the literature and meta-analysis did find a higher incidence of adverse effects (13% versus 7%) on phenytoin compared to Keppra, including worse neurological status and persistent fever [6]. No data had previously been reported comparing adverse effects or long-term outcomes for those on early Keppra seizure prophylaxis versus no treatment. We found no adverse effects in our retrospective cohort.

The evidence both for and against Keppra seizure prophylaxis suffers from several limitations. Foremost, there is no uniform protocol for Keppra prophylaxis. Dosages range in the literature from 500 mg twice daily to 1000 mg twice daily, with or without a 20 mg/kg to 1 g loading dose [3-4,9]. Within a single institution, the use of seizure prophylaxis may vary significantly [1]. Another major problem is that the frequency of seizure in this setting is fairly low, and so findings at a single institution may not reach statistical significance.

## Conclusions

Although a trend towards decreased seizures was seen in the Keppra prophylaxis group, this finding did not reach statistical significance. Further investigation is needed, and perhaps an ideal comparison would be done through a national quality database with a large enough cohort to compare Keppra prophylaxis to a control group.
